# Editorial: Opportunities and Challenges of Digitization in Rheumatology

**DOI:** 10.3389/fmed.2022.928361

**Published:** 2022-05-10

**Authors:** Philipp Sewerin

**Affiliations:** ^1^Rheumazentrum Ruhrgebiet Herne, Ruhr-University Bochum, Herne, Germany; ^2^Department and Hiller Research Unit of Rheumatology, Heinrich Heine University Düsseldorf, Universitaets-Klinik Duesseldorf, Düsseldorf, Germany

**Keywords:** digitization/digitisation, rheumatoid arthritis, psoriatic arthritis, systemic lupus erythematosus, telemedical applications

Today, digitization offers incredible opportunities for improving patient care in rheumatology. This applies to early diagnosis as well as therapy selection and monitoring. However, these opportunities are also accompanied by burdens and risks, which in some cases make the rapid use of such new technical achievements difficult or even impossible.

The topic “*Opportunities and Challenges of Digitization in Rheumatology*” precisely addresses these opportunities and risks and compiles exciting work with this focus in rheumatology.

In an exciting first paper, Lambrecht et al. examine the role of apps in patient care using the User Version of the Mobile Application Scale (uMARS). They can demonstrate that only a few applications meet the challenges on the user side and emphasize that this should be an important component in the evaluation of such tools.

In their work, the research group led by Song et al. examine the value and, above all, the challenges of telemedicine in rheumatology. They conclude that many challenges, such as data security or the reimbursement cannot always be met and call for additional recommendations on how to deal with telemedicine in rheumatology.

Pallua and Schirmer focus on quality needs in their work. They examine which standards have been defined so far and determine that these are necessary in addition to new therapy strategies to ensure and improve patient care at a high-quality level.

Giovannini et al. present a work entitled “The Digital Way to Intercept Psoriatic Arthritis.” In this paper, they examine the opportunities offered by digitalized medicine for the clinical picture of psoriatic arthritis. They state that modern technologies such as telemedicine, virtual visits, electronic health record, wearable technology, mobile health, artificial intelligence, and machine could shorten the diagnosis but also play an important role in the choice of the right therapy in the future.

Recker et al. investigate if ultrasound should be considered more in the education of students and support this with the results of their survey. They show that students are very interested in sonography and demand that it should be given a higher priority in the education of medical students.

The value of telemedicine in systemic lupus erythematosus is examined in the work of So et al. They note that patients respond and evaluate the use of telemedicine in SLE very different ways. For example, socioeconomic status was also critical for acceptance, which also poses new challenges on the part of the authors.

The research group led by Richter et al. focuses on the German pregnancy registry (Rhekiss) and examines the value of the registry's mobile app. They observe that the mobile app has been very well accepted and seems to be an important pillar, especially for PRO with younger patients.

Knevel et al. present the value of a Digital Diagnostic Decision Support Tool in their work, which was available online. They conclude that these online tools can help with early diagnosis, but that further optimization is necessary to enable and improve their use in everyday clinical practice.

All these papers do an excellent job of summarizing and critically evaluating the current discussion. They show very clearly that we will have to integrate telemedical procedures and digital processes into our algorithms in the future, but they also show that we must not do this without reflection. If this succeeds, digital processes can and will also be an important pillar in the diagnosis and therapy decision in rheumatology in the future. We must be open to accompany the implementation but also to critically question it again and again.

## Author Contributions

The author confirms being the sole contributor of this work and has approved it for publication.

## Conflict of Interest

The author declares that the research was conducted in the absence of any commercial or financial relationships that could be construed as a potential conflict of interest.

## Publisher's Note

All claims expressed in this article are solely those of the authors and do not necessarily represent those of their affiliated organizations, or those of the publisher, the editors and the reviewers. Any product that may be evaluated in this article, or claim that may be made by its manufacturer, is not guaranteed or endorsed by the publisher.

